# Femoral neck fractures in non-geriatric patients: femoral neck system versus cannulated cancellous screw

**DOI:** 10.1186/s12891-023-06140-3

**Published:** 2023-01-26

**Authors:** Guy Romeo Kenmegne, Chang Zou, Yue Fang, Xuanhong He, Yixiang Lin, Yijie Yin

**Affiliations:** 1 Trauma center, West China Hospital of Sichuan University, 610044 Chengdu, People’s Republic of China; 2 Department of Orthopedics, Orthopaedic Research Institute, West China Hospital, Sichuan University, Chengdu, People’s Republic of China

**Keywords:** Cannulated cancellous screw, Femoral neck fracture, Femoral neck system

## Abstract

**Background:**

The fractures of femoral neck account for 50% among hip fractures with around 3%-10% occurring in younger population of below 65 years. The newly introduced FNS as management approach appears to be a potential alternative to the traditional CCS. The aim of this study was to compare the clinical efficacy and outcome of the femoral neck system (FNS) and the cannulated cancellous screw (CCS) in the treatment of femoral neck fractures in adult below 65 years of age.

**Methods:**

Data of 114 patients between 18–65 years, admitted in our department for femoral neck fracture from January 2019 to March 2021 were retrospectively studied and ranged into two groups based on the surgical methods: FNS group (56 patients) and CCS group (58 patients). The variables of interest including demographic and clinical variables (age, gender, fracture mechanism, injury side and classification), perioperative parameters(operation time, intraoperative bleeding, incision length and hospitalization time), postoperative outcomes and complications (fracture healing time, nonunion rate, femoral neck avascular necrosis, aseptic screw loosening and the Harris Hip Score), were analyzed and compared between the two groups.

**Results:**

All 114 patients presented satisfactory reduction and were followed-up for a period of 12 to 36 months (mean 27 ± 2.07 months); there were no significant differences between both groups in terms of age, gender, fracture classification, side of injury, mechanism of injury, the operative time, intraoperative blood loss and the hospital length of stay. However, the fracture healing time between FNS group and CCS group was statistically significant (*p* < 0.05), respectively 2.86 ± 0.77 and 5.10 ± 0.81 months. The significant differences were also found in terms of numbers of fluoroscopies 8.34 ± 1.38 Vs 17.72 ± 2.19, the HHS 87.80 ± 1.92 Vs 84.28 ± 2.24, postoperative complications 8 (14.28%) Vs 26 (44.82) respectively in FNS and CCS group.

**Conclusion:**

FNS presented satisfactory outcomes had significantly lower complications rate, therefore, can be one of the alternatives for internal implantation devices in treatment of femoral neck fracture in non-geriatric population.

## Introduction

The fractures of femoral neck account for 50% among hip fractures with around 3%-10% occurring in younger population of below 65 years [1–3]; therefore, they are considered as a major public health concern. Considering factors such as population growth and ageing, the incidence of femoral neck fractures (which was 1.6 million people per year in 1990) is projected to increase to 2.6 million in 2025 and 6.3–6.5 million by the year 2050 with serious socioeconomic impact, and significant challenges to orthopedic surgeons [4–6].

While in elderly population the femoral neck fracture is due to low energy force, in younger population it is mostly due to high energy trauma such as fall from the height or Road traffic accidents [7, 8]. There have been an estimated 20–30% incidence rate of complications in patients after femoral neck fracture among which avascular necrosis, implant failure, nonunion and malunion are the most incriminated [9]. The surgical management and the implant selection is still a challenging task among orthopedic specialist. The cannulated cancellous screw (CCS) and the Dynamic Hip Screw (DHS) are widely used. The CCS presents some advantages as it provides the torsional stability and preservation of blood vessels in the femoral head and neck. However it has limitation in overcoming the vertical shear instability encountered in Pauwels type III femoral neck fracture in which case the DHS is the best alternative [10–12]. The femoral neck system (FNS), designed by the Association for the Study of Internal Fixation and DePuy Synthes combines the advantages of both cannulated screw and DHS [13]. Some authors have recently reported important findings regarding the mechanic and the biomechanical stability of the internal fixation using FNS in unstable femoral neck fractures; according to the authors, the Placement of the bolt tip of FNS at proximity and inferiorly to the subchondral bone in pauwels III femoral neck fractures respectively provided higher stability and increased inter-fragmentary sliding distance; additional 5 mm gap between the diaphysis and the plate probably adjust the depth of the bolt [10].

The clinical efficacy and postoperative outcome of internal fixation using FNS compared to that of the traditional cannulated cancellous screw remained not very well documented. This study aimed to compare the clinical efficacy and postoperative outcome between FNS and CCS in the treatment of femoral neck fractures in population of below 65 years of age.

## Methods

We conducted a retrospective study of patients between 18–65 years, admitted in our department for femoral neck fracture from January 2019 to March 2021. The inclusion criteria were as follow: (1) non geriatric patients (below 65 years), (2) imaging diagnosis classification of femoral neck fracture (Garden and Pauwels), (3) fracture treated with the femoral neck system and (4) fracture treated with cannulated cancellous screw. The exclusion criteria were: (1) fracture treated with conservative management, (2) poly-trauma patients (3), patients managed with any other surgical method apart from the above mentioned two methods, (4) patients with any other particular medical history, chronic alcohol abusers, (5) patients with Traumatic Brain Injury (TBI) or Glasgow Coma Scale score of < 14, and 6) patients with pathological fracture and a minimum follow-up period of 12 months. A total of 147 were retrospectively studied. Overall, 114 out of 147 patients met the inclusion criteria. They were divided into two groups: patients managed with FNS (*n* = 56) and those treated with CCS (*n* = 58).

All the patients included in our study had their operation done as soon as possible to reduce the chances of perioperative complications such as pneumonia, Deep Venous Thromboembolism (DVT). The variables of interest included demographic variables (gender and age), clinical variables (mechanism of injury, fracture type, and the side of injury), perioperative parameters (such as operation time, intraoperative bleeding, incision length and hospital length of stay), postoperative outcomes and complications (fracture healing time, nonunion rate, femoral neck avascular necrosis, aseptic screw loosening and the Harris Hip Score). These variables were compared between the two populations groups.

Patients’ data were collected following the anonymous method, contact numbers and personal identity completely erased for ethical consideration. The informed consent was obtained from all the patients and the study was approved by the institutional review board and the Ethic committee of our institution.

### Surgical procedure and steps

After successful anesthesia, the patient was placed on the orthopedic lower extremity traction bed, in supine position. The C-arm fluoroscopy was placed in between the patient’s lower limbs to allow easy taking of anteroposterior and lateral images. Adjusted with traction, an adduction and internal rotation of the lower extremity was performed to reduce the fracture end. This maneuver was repeated and monitored under the C-arm fluoroscopy to confirm the fracture alignment. After the reduction was satisfactory, a routine preoperative skin preparation, asepsis protocol and draping was done on the operation field.

A longitudinal incision of about 3 to 5 cm in length was made on the plane of the lateral lesser trochanter of the hip. A blunt dissection of the underlying subcutaneous tissue, deep fascia layer up to the bone cortex was performed with the use of clamp forceps to avoid the contusion of the tensor fascia lata. Through the tensor fascia lata muscle fiber gap, the incision can reach the lower edge of the femoral tuberosity,where it can then be peelled down.

**Fig. 1:**
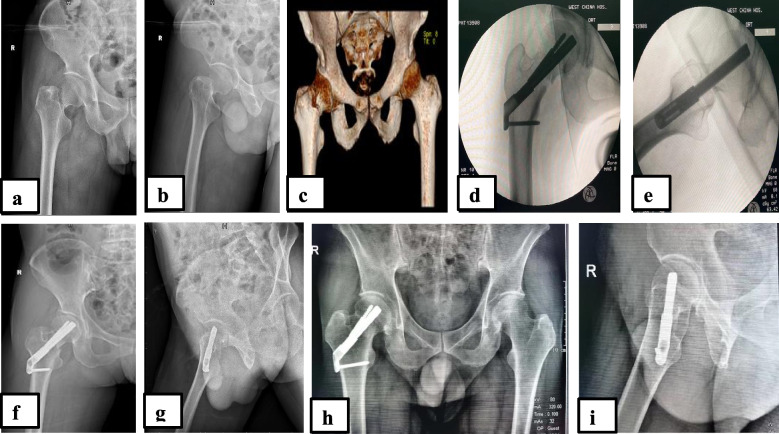
A 60 years old male patient with right femoral neck fracture (Garden type III), managed operatively with internal fixation using Femoral Neck System 24 h after injury. Preoperative radiographs anteroposterior view (**a**), lateral view (**b**) and 3D CT scan (**c**); intraoperative fluoroscopic anteroposterior view (**d**), lateral view (**e**); immediate postoperative radiographs anteroposterior view (**f**) and lateral view (**g**); postoperative 13 months radiographs anteroposterior view (**h**) and lateral view (**i**)

In FNS procedure, a 2.5 mm diameter Kirshner wire was inserted through the tip of the greater trochanter up to the femoral head to prevent the rotation of the femoral head in its socket during the guide pin or FNS implant penetration into the femoral neck; afterwards a guide pin was inserted into the inferior cortex of the femoral neck. Under C-arm fluoroscopy guidance, the FNS system was placed from below the greater trochanter through the femoral neck, then to the femoral head. Thereafter, a locking screw was placed to fix the plate in the femur through the lesser trochanter. The central nail was inserted into the main nail, to provide a firm compression force (Fig. 1).

**Fig. 2:**
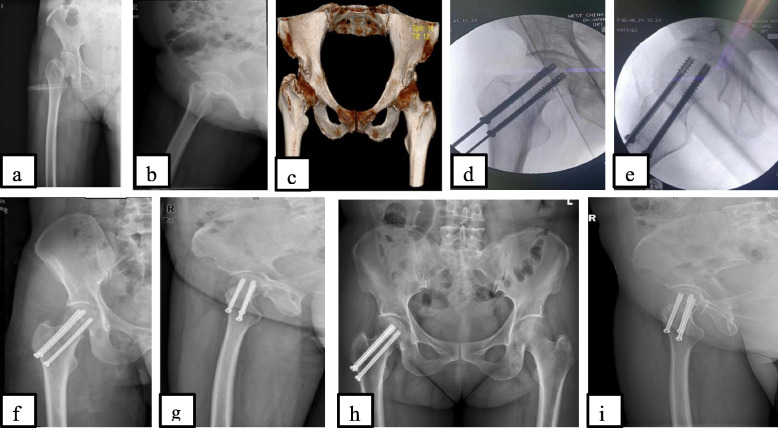
A 48 years old female patient who sustained a femoral neck fracture garden III after a fall, managed operatively with cannulated cancellous screw 24 h after injury. Preoperative X-ray anteroposterior view (**a**), lateral view (**b**) and 3D CT scan (**c**); intraoperative fluoroscopic images anteroposterior view (**d**), lateral view (**e**); immediate postoperative X-ray anteroposterior view (**f**) and lateral view (**g**); postoperative 12 months radiographs anteroposterior view (**h**) and lateral view (**i**)

While in CCS, the 2.5 mm K-wire was inserted at the middle to lower 1/3 of the femoral neck; 3 parallel guide wires were inserted simultaneously in the femoral neck through the head. As the guide pins were inserted through the femoral head and neck, the fluoroscopy was used to determine their appropriate direction and length. The 3 screws were inserted simultaneously in a way to obtain an inverted triangle with the apex oriented downward and the base upward (Fig. 2).

The fracture reduction was visualized on the fluoroscopy to appreciate the position of the internal fixator as well as the fracture reduction (Figs. 1d,e and 2d,e). It should be remembered that, in cases where closed reduction was impossible, opened reduction was an option via anterior approach with Smith-Peterson incision followed by a T-shape incision on the joint capsule to expose the fractured end and attend the reduction under direct vision.

### Perioperative management

All included patients received prophylactic anticoagulant therapy with oral rivaroxaban 10 mg/day. The anticoagulant prophylaxis was discontinued 24 h prior to the operation and was continued for 3 weeks after the discharge from the hospital in order to prevent the DVT. Before induction of anesthesia (30 min prior to skin incision), all the included patients received an intravenous prophylactic first generation cephalosporin cefazolin; the prophylactic antibiotic was discontinued continued 24 h postoperatively. This was accompanied with an intravenous 1 g of tranexamic acid (TXA) with a repeated dose 6 h postoperatively. The mobilization of the patient was ordered on postoperative day 1 with partial weight bearing in patients with stable fracture, however this weight bearing was delayed in patients with unstable fracture (as these had higher risk of vascular damage related to the initial injury itself) in whom quadriceps femoris exercise, hip, knee and ankle passive and active exercise were recommended in order to minimize edema in the lower limb. Loxoprofen sodium tablet 180 mg/day in 3 divided doses was recommended for 3 weeks after discharge for postoperative pain control. The outpatient follow-up was recommended to all patients every 3 months up to one year and every semester afterwards.

### Outcome measurement

We meticulously and carefully collected, assessed and recorded all clinical data of the included patients such as (1) the operation time, (2) the intraoperative blood loss, (3) the incision length/size, (4) the hospital length of stay (LOS), (5) the postoperative complications including nonunion rate; femoral neck avascular necrosis; aseptic screw loosening (usually identified if there were any signs of screw penetration or withdrawal on plain radiographs) and the postoperative femoral neck shortening, (6) the fracture healing time, considered as time required to observe radiological evidence of healing on plain radiograph, accompanied by absence of percussion pain in the hip joint or the lower limb in the affected side: this time was previously defined within an average of three months in most patients according to the literature [14], (7) the Harris hip score (HHS). On the questionnaire-base, the Hip function of each patients were assessed in outpatient clinic on last follow-up using the translated version of the HHS guideline published in 1969 which takes into consideration the patient’s pain (44 points), the mobility and walking (47 points), range of motion ROM (5 points), and absence of deformities (4 points) with the total score of 100 ranging from extreme symptoms (HHS = 0) and no symptoms (HHS = 100); accordingly, scores above 90 were considered excellent, score between 80 and 90 were considered as good results, those between 70 and 80 were defined as fair results, while values below 70 were defined as poor outcome [3, 15, 16]. The femoral head avascular necrosis and nonunion (at one year after operation) were assessed using plain radiographs. On full length leg radiographic images, the full length of the affected leg compared to the non-affected side was obtained and evaluated to detect the presence of shortening; The degree of the femoral neck shortening was classified as none or mild, moderate and severe, when the extent of shortening was (< 5 mm); (5–10 mm) and (> 10 mm) respectively [17] and patients were diagnosed as shortened femoral neck if shortening was above 10 mm.

The femoral head shortening was measured on standard X-ray using the same method as described by Haider et al. [3]. We measured the center–collum–diaphysis (CCD) angle to determine the length of the femoral neck and the distance joining the center of the femoral head to the inferior end of the lesser trochanter. We compared the finding to the contralateral side. Additionally, a plain radiograph of the full limb was obtained and the full leg distance was measured accordingly. The numbers of intraoperative fluoroscopies was retrieved and recorded from the operation records documented in each patient’s file.

### Statistical analysis

Continuous variables such as, operation time, intraoperative blood loss, length of the incision, age, hospital Length of stay (LOS) and number of fluoroscopies were expressed as mean and standard deviation (SD), then analyzed using the Student’s t test. The chi-square test was used for categorical variables analysis. The statistical analyses were done using SPSS 20.0 (SPSS, IBM, USA). A value of *P* < 0.05 was considered as statistically significant.

## Results

A total of 114 patients aged below 65 years, operated with FNS method (56 patients) and CCS method (58 patients) with a follow-up period of 12 to 36 months (mean 27 ± 2.07 months) were included and respectively studied; the results of the analyses are presented as follows:

There was non-significant gender difference between both groups (*p* = 0.46) while there was a significant age difference between both groups (*p* < 0.001). The Garden classification types III was the most encountered in both groups. The Pauwels classification type III was the most encountered in FNS group (57.14%); the difference of distribution between both groups was statistically significant for Garden classification (*p* = 0.02) as well as Pauwels classification (*p* < 0.01) (Table 1).

**Table 1:** Patient’s demographics distribution and fracture characteristics

Variables		FNS group	CCS group	*P –value*
Gender n (%)	Male	29(51.78)	34(58.62)	0.46
	female	27(48.21)	24(41.37)	
Age (years)		58.20 ± 15.15	40.45 ± 16.57	< 0.001
Mechanism of injury n (%)	Fall from height	47(83.93)	41(70.68)	0.22
	Road traffic accident	4(7.14)	7(12.06)	
	Others	5(8.93)	10(17.24)	
Side of injury n (%)	Right	26(46.43)	26(44.82)	0.83
	Left	30(53.57)	32(55.17)	
Garden classification n (%)	I	6(10.71)	0	0.02
	II	13(23.21)	12(20.68)	
	III	37(66.07)	41(70.68)	
	IV	0	5(8.62)	
Pauwels classification n (%)	Type I	8 (14.28)	15(25.86)	< 0.01
	Type II	16 (28.57)	29(50.00)	
	Type III	32 (57.14)	14(24.13)	

The difference in number of fluoroscopies and incision length between both groups was statistically significant (*p* < 0.001). There was no significant difference in terms of operation time (55.11 ± 30.22 min in FNS group and 64.19 ± 29.52 min in CCS group), (*p* = 0.05). The volume of intraoperative blood loss (35.71 ± 29.66 ml in FNS group and 35.69 ± 29.15 ml in CCS group), as well as the hospital length of stay (8.64 ± 3.99 days in FNS group and 9.36 ± 4.22 days in CCS group) showed nonsignificant difference (*p* = 0.99 and *p* = 0.65 respectively), (Table 2).

**Table 2:** Operative parameters

Variables	FNS group (mean ± SD)	CCS group (mean ± SD)	*P -value*
Number of fluoroscopies (n)	8.34 ± 1.38	17.72 ± 2.19	< 0.001
Operation time (min)	55.11 ± 30.22	64.19 ± 29.52	0.05
Intraoperative blood loss (ml)	35.71 ± 29.66	35.69 ± 29.15	0.99
Incision length (cm)	4.86 ± 1.19	3.88 ± 1.03	< 0.001
Hospital Length of Stay (days)	8.64 ± 3.99	9.36 ± 4.22	0.65

The fracture healing time was significantly shorter (2.86 ± 0.77 months) in FNS group than in CCS group (5.10 ± 0.81 months), (*p* < 0.01).

Overall, there was a statistical difference in terms of postoperative complications in both groups: 14.28%(8/56) in the FNS group, which was lower than 44.82%(26/58) in the CCS group (*p* < 0.001).

The evaluation of the HHS demonstrated a significant difference between FNS group (87.80 ± 1.92) and CCS group (84.28 ± 2.24) with *p* = 0.04 (Table 3).

**Table 3:** Postoperative follow-up and complications

Variables	FNS group	CCS group	*P -value*
Fracture healing (months)	2.86±0.77	5.1±0.81	<0.001
Harris Hip Score (HHS)	87.80±1.92	84.28±2.24	0.04
Complications n (%)	8(14.28)	26(44.82)	<0.001
Non-union n (%)	2(3.57)	5(8.62)	
Femoral neck avascular necrosis n (%)	1(1.79)	4(6.89)	
Aseptic Screw loosening n (%)	2(3.57)	5(8.62)	
Femoral neck shortening n (%)	3(5.35)	12(20.68)	

## Discussion

The incidence of adult femoral neck fracture had been significantly increased proportionately with the increase of high energy trauma such as road traffic accident, fall from height [18]. The treatment of femoral neck fracture in elderly is dominated by the hip arthroplasty; while in younger patients (below 65 years), the current approach consists of minimally invasive technique using close reduction and internal fixation [19, 20]. The traditional existing methods of internal fixation include anteromedial femoral neck plate with cannulated screws, Hansson pin system, Dynamic Hip Screw (DHS), cannulated screw, FNS, etc. [1, 9, 11, 17, 21].

The use of CCS in the fixation of femoral neck fracture in younger population has been widely documented; minimally invasive, it offers anti-rotation properties, low cost and convenient for most femoral neck fractures [1, 13]. However, for Pauwels type III fracture, the biomechanical properties of the cannulated screws are poor [22, 23]. The CCS which consists of 3 screws inserted in the configuration of an inverted triangle provides a torsional stability and preservation of blood vessels in the femoral head and neck. Its limitation in overcoming the vertical shear instability encountered in Pauwels type III femoral neck fracture has given to DHS the priority in this patient group [10–12]. The FNS mechanically combines the properties of both CCS and DHS [13]. Other scholars analyzed the biomechanical properties of FNS and concluded that, it was a reliable method for internal fixation of displaced femoral neck fractures [24, 25].

The placement and adjustment of screw in the CCS group requires multiple fluoroscopies images, and longer operation time, consequently more exposure of the patient and staff to the radiations. In our study, the number of fluoroscopies was 8.34 ± 1.38 in FNS group and 17.72 ± 2.19 in CCS group (*p* < 0.001). The operation time was 55.11 ± 30.22 min in FNS group and 64.19 ± 29.52 min in CCS group (*p* = 0.05). He et al. [26] reported the mean number of fluoroscopies in FNS 10.58 ± 1.89 Vs 18.33 ± 3.82 in CCS (*p* < 0.001); with a mean operation duration of 49.94 ± 14.46 min in FNS Vs 56.11 ± 12.48 min in CCS (*p* = 0.06). We believe that shorter duration of operation and a reduced number of fluoroscopies are beneficial to the patient.

The basic data analyses in our study reported a significant difference in patients’ ages, and the fracture classification in the two groups; however, we failed to relate these differences to the choice of the treatment approaches as well as their final outcomes, moreover, we have not found consistent studies in the literature discussing similar findings. The postoperative complications following the fracture of femoral neck are well documented in literature. This involving: fracture nonunion, avascular necrosis of the femoral neck, neck shortening and the loosening of the internal fixator.

In most patients, the reasonable time to expect union is 3 months or longer [14]; however, although the reduction and fixation of the femoral head is intended to restore the blood supply to the fractured femoral neck, overall 10–20% and 10–30% of cases will still develop aseptic nonunion and femoral neck avascular necrosis respectively [14, 27]; these consisting the main causes of fracture revision with hip arthroplasty [27]. In our study, the fracture healing time was 2.86 ± 0.77 months in FNS group and 5.10 ± 0.81 months in CCS group, (*p* < 0.001) with the non-union rate of 3.57% in FNS group and 8.62% in CCS group. The femoral neck avascular necrosis was encountered in 1.79% of cases in FNS group and 6.89% of cases in CCS group. The aseptic screw loosening was found in 3.57% and 8.62% of cases respectively in FNS group and in CCS group.

Hu et al. [7] reported an incidence of 10% nonunion in FNS and 12.5% in CCS (*p* = 0.79); a 5% rate of femoral head necrosis in FNS and 12.5% in CCS (*p* = 0.38); 0% of screw loosening (screw cut-out) in FNS and 50% in CCS, (*p* = 0.01). Tang et al. [18] reported 4% nonunion, 2% of femoral neck necrosis and 6% screw loosening in FNS group; moreover, the authors revealed 9% nonunion, 7% femoral neck necrosis and 11% screw loosening in CCS. Zhou et al. [13] reported a higher incidence of complications in CCS (30%) than in FNS (6.7%), (*p* = 0.04). He et al. [1] reported an incidence of complications of 6.1% (2/33) in FNS group and 25% (9/36) in CCS group (*P* = 0.03). Herein, our overall complication rate was 14.28% in FNS group and 44.82% in CCS group, which was higher than in the above previous studies; however, we noticed that this was mainly related to the high incidence of neck shortening in both groups.

Independently to the treatment approach, there is a certain extent of femoral neck shortening (50%–60%), but whether this shortening will lead to an impairment of the hip function remain controversial [17, 28]. One scholar reported that, femoral neck shortening following femoral neck fracture fixation is a common complication with a rate of 30%-31%; additionally, the authors supported that a shortening of even 5 mm can cause limping [17]. Felton et al. [29] reported no/mild shortening (≤ 5 mm) in 2/3^rd^ of patients and moderate/severe (> 10 mm) in 1/3^rd^ of patients with femoral neck fracture managed operatively; furthermore, the authors reported that the hip function worsened with the severity of the shortness. Sahin et al. [8] found a significant shortness of femoral neck (9.5 ± 5.9 mm) in patients treated with CCS. Zlowodzki et al. [17] reported 2/3^rd^ (66%) healing with shortening (> 5 mm) in hip fracture treated with multiple CCS. Shu et al. [21] presented a significant shortening (> 5 mm) of femoral neck following surgery in patients treated with CCS and dynamic compression locking system (DCLS), moreover, the authors reported that DCLS shortening in the horizontal (4.4 ± 1.45 mm in DCLS Vs 4.4 ± 1.45 mm in CCS) and vertical direction (6.8 ± 2.27 mm in DCLS Vs 6.8 ± 2.27 mm) were significantly lower than those in the CCS group (*P* = 0.00). Zlowodski et al. [30] in another study reported 34% of no/mild shortening, 36% of moderate shortening, and 30% severe shortening in femoral neck fractures patients treated with CCS. In the current study, the findings showed a higher incidence of femoral neck shortening in CCS group compared to FNS group. According to previous authors, there is always a certain extent of shortening following femoral neck fracture regardless the choice of the operative approach; therefore, in our study, we considered patients to be diagnosed as shortened femoral neck when shortening was severe (more than 10 mm); they were 3(5.35%) in FNS group and 12(20.68%) in CCS group.

The findings of the biomechanical experiments reported by Stoffel et al. [25] together with the results of our outcomes suggested that the construct of FNS plays an important role in resisting femoral neck shortening. This result was basically consistent with those found in previous literature [1, 7, 18].

The HHS is an important tool used by clinicians for the evaluation of hip function. He et al. [1] reported Harris Hip Score in the FNS group as 90.42 ± 4.82 and 88.44 ± 5.91 in the CCS group (*p* = 0.13). Hu et al. [7] found 85.90 ± 5.98 in FNS compared to 81.92 ± 8.34 in CCS group. Zhou et al. [13] reported Harris score of 86.16 ± 7.26 in the FNS group comparing to (82.37 7.52) in the cannulated screw group (*P* = 0.039). In this study, the HHS were 87.80 ± 1.92 and 84.28 ± 2.24, respectively in FNS group and CCS; there was minimal clinically important difference between both groups (*p* = 0.04). These results were consistent with the above studies [1, 7, 13] and these values found in our study were considered as good results according to the current literature [13, 31].

The Vascular complication such as DVT is well documented in patients with hip fracture and it is usually prevented with the prophylactic use of Low molecular weight heparin [2]. In our study, all the patients received perioperative oral rivaroxaban and 0 case of complications related to DVT was identified.

Our study presents some limitations: firstly, its retrospective nature makes it susceptible to possible minor errors as the patients were not randomly assigned. Secondly, although efforts for standardizing shortening measurements were made, we assume they might have been affected by multiple rotational alignment of the femur on digital radiographs. Lastly, in our study, the fixation approach and the implant selection was chosen based on our own clinical experience, we acknowledge that this could cause possible bias in our results. Additionally, the difference regarding the individuals’ ages and fracture classification which could not be associated to any outcome or findings constituted another shortcoming to this study: We therefore acknowledge that a randomized control trial is needed to further authenticate our findings.

## Conclusion

In conclusion, both FNS and CCS methods present comparable operation time, intraoperative blood loss but significant difference in fracture healing time, numbers of intraoperative fluoroscopies and length of incision. Furthermore, there was satisfactory functional outcome with slightly higher HHS and lower incidence of complications in FNS group compared to CCS group, therefore, FNS can be one of the alternatives for internal implantation devices in treatment of femoral neck fracture in non-geriatric population.

## Data Availability

The datasets used and/or analyzed during the current study are available from the corresponding author on reasonable request.

## References

[1] He C, Lu Y, Wang Q, Ren C, Li M, Yang M, Xu Y, Li Z, Zhang K, Ma T (2021). Comparison of the clinical efficacy of a femoral neck system versus cannulated screws in the treatment of femoral neck fracture in young adults. BMC Musculoskelet Disord.

[2] Pauyo T, Drager J, Albers A, Harvey EJ (2014). Management of femoral neck fractures in the young patient: A critical analysis review. World journal of orthopedics.

[3] Haider T, Schnabel J, Hochpöchler J, Wozasek GE (2018). Femoral shortening does not impair functional outcome after internal fixation of femoral neck fractures in non-geriatric patients. Arch Orthop Trauma Surg.

[4] Gullberg B, Johnell O, Kanis JA (1997). World-wide projections for hip fracture. Osteoporos Int.

[5] Parker MJ (2000). The management of intracapsular fractures of the proximal femur. The Journal of bone and joint surgery British.

[6] Zielinski SM, Keijsers NL, Praet SF, Heetveld MJ, Bhandari M, Wilssens JP, Patka P, Van Lieshout EM (2013). Femoral neck shortening after internal fixation of a femoral neck fracture. Orthopedics.

[7] Hu H, Cheng J, Feng M, Gao Z, Wu J, Lu S (2021). Clinical outcome of femoral neck system versus cannulated compression screws for fixation of femoral neck fracture in younger patients. J Orthop Surg Res.

[8] Şahin A, Agar A, Gülabi D, Ertürk C (2020). Comparison of dynamic hip screw and antirotation screw with cannulated screw in the treatment of transcervical collum femoris fractures. Joint diseases and related surgery.

[9] Slobogean GP, Stockton DJ, Zeng B, Wang D, Ma BT, Pollak AN (2017). Femoral Neck Fractures in Adults Treated With Internal Fixation: A Prospective Multicenter Chinese Cohort. J Am Acad Orthop Surg.

[10] Jung CH, Cha Y, Yoon HS, Park CH, Yoo JI, Kim JT, Jeon Y (2022). Mechanical effects of surgical variations in the femoral neck system on Pauwels type III femoral neck fracture : a finite element analysis. Bone & joint research.

[11] Cha YH, Yoo JI, Hwang SY, Kim KJ, Kim HY, Choy WS, Hwang SC (2019). Biomechanical Evaluation of Internal Fixation of Pauwels Type III Femoral Neck Fractures: A Systematic Review of Various Fixation Methods. Clin Orthop Surg.

[12] Bliven E, Sandriesser S, Augat P, von Rüden C, Hackl S (2020). Biomechanical evaluation of locked plating fixation for unstable femoral neck fractures. Bone & joint research.

[13] Zhou XQ, Li ZQ, Xu RJ, She YS, Zhang XX, Chen GX, Yu X (2021). Comparison of Early Clinical Results for Femoral Neck System and Cannulated Screws in the Treatment of Unstable Femoral Neck Fractures. Orthop Surg.

[14] Angelini M, McKee MD, Waddell JP, Haidukewych G, Schemitsch EH (2009). Salvage of failed hip fracture fixation. J Orthop Trauma.

[15] Josipović P, Moharič M, Salamon D (2020). Translation, cross-cultural adaptation and validation of the Slovenian version of Harris Hip Score. Health Qual Life Outcomes.

[16] Ahmed MIR, Prashanth KRT (2020). Osteosynthesis in Neglected Femoral Neck Fracture in an Adolescent by Internal Fixation Alone - A Case Report. Journal of orthopaedic case reports.

[17] Zlowodzki M, Brink O, Switzer J, Wingerter S, Woodall J, Petrisor BA, Kregor PJ, Bruinsma DR, Bhandari M (2008). The effect of shortening and varus collapse of the femoral neck on function after fixation of intracapsular fracture of the hip: a multi-centre cohort study. The Journal of bone and joint surgery British.

[18] Tang Y, Zhang Z, Wang L, Xiong W, Fang Q, Wang G (2021). Femoral neck system versus inverted cannulated cancellous screw for the treatment of femoral neck fractures in adults: a preliminary comparative study. J Orthop Surg Res.

[19] Samsami S, Saberi S, Sadighi S, Rouhi G (2015). Comparison of Three Fixation Methods for Femoral Neck Fracture in Young Adults: Experimental and Numerical Investigations. Journal of medical and biological engineering.

[20] Zhuang L, Wang L, Xu D, Wang Z (2019). Anteromedial femoral neck plate with cannulated screws for the treatment of irreducible displaced femoral neck fracture in young patients: a preliminary study. European journal of trauma and emergency surgery : official publication of the European Trauma Society.

[21] Shu DP, Xiao YP, Bei MJ, Ji T, Peng YJ, Ma B, Li SG (2020). Dynamic compression locking system versus multiple cannulated compression screw for the treatment of femoral neck fractures: a comparative study. BMC Musculoskelet Disord.

[22] Ye Y, Chen K, Tian K, Li W, Mauffrey C, Hak DJ (2017). Medial buttress plate augmentation of cannulated screw fixation in vertically unstable femoral neck fractures: Surgical technique and preliminary results. Injury.

[23] Li J, Zhao Z, Yin P, Zhang L, Tang P (2019). Comparison of three different internal fixation implants in treatment of femoral neck fracture-a finite element analysis. J Orthop Surg Res.

[24] Schopper C, Zderic I, Menze J, Müller D, Rocci M, Knobe M, Shoda E, Richards G, Gueorguiev B, Stoffel K (2020). Higher stability and more predictive fixation with the Femoral Neck System versus Hansson Pins in femoral neck fractures Pauwels II. Journal of orthopaedic translation.

[25] Stoffel K, Zderic I, Gras F, Sommer C, Eberli U, Mueller D, Oswald M, Gueorguiev B (2017). Biomechanical Evaluation of the Femoral Neck System in Unstable Pauwels III Femoral Neck Fractures: A Comparison with the Dynamic Hip Screw and Cannulated Screws. J Orthop Trauma.

[26] Dhar SA, Gani NU, Butt MF, Farooq M, Mir MR (2008). Delayed union of an operated fracture of the femoral neck. Journal of orthopaedics and traumatology : official journal of the Italian Society of Orthopaedics and Traumatology.

[27] Haidukewych GJ, Rothwell WS, Jacofsky DJ, Torchia ME, Berry DJ (2004). Operative treatment of femoral neck fractures in patients between the ages of fifteen and fifty years. The Journal of bone and joint surgery American.

[28] Stockton DJ, Lefaivre KA, Deakin DE, Osterhoff G, Yamada A, Broekhuyse HM (2015). OʼBrien PJ, Slobogean GP: Incidence, Magnitude, and Predictors of Shortening in Young Femoral Neck Fractures. J Orthop Trauma.

[29] Felton J, Slobogean GP, Jackson SS, Della Rocca GJ, Liew S, Haverlag R, Jeray KJ, Sprague SA, OʼHara NN, Swiontkowski M, et al. Femoral Neck Shortening After Hip Fracture Fixation Is Associated With Inferior Hip Function: Results From the FAITH Trial. J Orthop Trauma. 2019;33(10):487–96. 10.1097/BOT.0000000000001551 31464855

[30] Zlowodzki M, Ayeni O, Petrisor BA, Bhandari M (2008). Femoral neck shortening after fracture fixation with multiple cancellous screws: incidence and effect on function. J Trauma.

[31] Kalairajah Y, Azurza K, Hulme C, Molloy S, Drabu KJ (2005). Health outcome measures in the evaluation of total hip arthroplasties–a comparison between the Harris hip score and the Oxford hip score. J Arthroplasty.

